# A randomized, double-masked, placebo-controlled crossover trial on the effects of L-ornithine on salivary cortisol and feelings of fatigue of flushers the morning after alcohol consumption

**DOI:** 10.1186/1751-0759-7-6

**Published:** 2013-02-18

**Authors:** Takeshi Kokubo, Emiko Ikeshima, Takayoshi Kirisako, Yutaka Miura, Masahisa Horiuchi, Akira Tsuda

**Affiliations:** 1 Developmental Research Group, Functional Food Business Project, Kirin Holdings Company, Limited, 1-13-5 Fukuura Kanazawa-ku, Yokohama 236-0004, Japan; 2 Department of Environmental Medicine, Kagoshima University Graduate School of Medical and Dental Sciences, Kagoshima University, 8-35-1, Sakuragaoka, Kagoshima, 890-8544, Japan; 3 Graduate School of Psychology, Kurume University, Miimachi Kurume, Fukuoka, 839-8502, Japan

**Keywords:** Ornithine, Residual alcohol effects, Salivary cortisol, Flusher

## Abstract

**Background:**

Residual alcohol effects on physiological and psychological symptoms are commonly experienced the morning after alcohol consumption. The purpose of this study was to assess the effects of L-ornithine on subjective feelings and salivary stress markers the morning after alcohol consumption and to investigate whether L-ornithine acutely accelerates ethanol metabolism.

**Methods:**

This study had a randomized, placebo-controlled, double-masked crossover design. Subjects were all healthy Japanese adults with the ‘flusher’ phenotype for alcohol tolerance. In experiment 1, 11 subjects drank 0.4 g/kg body weight alcohol 1.5 h before their usual bedtime. Half an hour after drinking, they ingested either a placebo or 400 mg ornithine. The next morning on awakening, subjects completed a questionnaire containing a visual analog scale (VAS), the Oguri-Shirakawa-Azumi sleep inventory MA version (OSA-MA), and a profile of mood states (POMS) and collected a saliva sample for measurement of salivary stress markers (cortisol, secretory immunoglobulin A, and α-amylase). In experiment 2, placebo or 400 mg ornithine were administrated to 16 subjects both before and after drinking, and the feeling of drunkenness, breath ethanol concentration and one-leg standing time were repeatedly investigated until 180 min after alcohol consumption.

**Results:**

There were significant decreases in “awareness”, “feeling of fatigue” and “lassitude” VAS scores and in “anger-hostility” and “confusion” POMS scores and a significant increase in “sleep length” in the OSA-MA test. Salivary cortisol concentrations on awakening were reduced after ornithine supplementation. There were no differences between ornithine and placebo in any of the subjective or physiological parameters of acute alcohol metabolism.

**Conclusions:**

Taking 400 mg ornithine after alcohol consumption improved various negative feelings and decreased the salivary stress marker cortisol the next morning. These effects were not caused by an increase in acute alcohol metabolism.

## Background

L-ornithine is an amino acid that is not incorporated in proteins but functions in the urea cycle in the liver. The urea cycle converts ammonia, a harmful nitrogen metabolite, into urea. Foods like corbiculidae clams, tuna, and cheese contain high amounts of ornithine [1-3]. Administration of ornithine is well known to stimulate the urea cycle [4,5]. In pharmacological therapy, ornithine is used to decrease blood ammonia concentrations and reduce the symptoms of hepatic encephalopathy associated with liver cirrhosis [6]. In addition, ornithine improves the subjective feeling of fatigue in patients with hepatic disease or evoked by exercise [7,8]. Alcohol consumption transiently increases blood ammonia levels [9,10]. Both ammonia and alcohol are toxic and mainly metabolized in the liver, and there is some evidence that their respective metabolisms influence each other [11]. Alcohol dehydrogenase, which metabolizes ethanol to acetaldehyde, and aldehyde dehydrogenase (ALDH), which metabolizes acetaldehyde to acetic acid, play pivotal roles in degrading alcohol. The gene encoding ALDH2 is a major gene determining alcohol sensitivity and drinking behavior, particularly in Asian people including Japanese [12]. People who are heterozygous for wild-type and mutated ALDH2 are classified as ‘flushers’. Flushers have very weak ALDH2 activity and show rapid flushing of the skin at comparatively low doses of alcohol owing to the accumulation of acetaldehyde in the body. In contrast, non-flushers are homozygous for wild type ALDH2 and have normal alcohol metabolism.

Although moderate use of alcohol has some beneficial aspects, excessive consumption of alcohol is undoubtedly harmful to the health. Alcohol also affects psychological and physiological states. Relatively low consumption of alcohol can shorten the initiation of sleep and promote good moods such as conviviality or a general pleasant feeling. Conversely, high consumption of alcohol increases nocturnal awakening and may bring about bad feelings, anxiety, or depression. Flushers in particular tend to suffer from these negative effects due to their low ability to metabolize alcohol. Thus, simple and convenient methods for reducing the harmful effects of alcohol will be beneficial.

We previously reported that 800 mg L-ornithine-L-aspartate, a stable salt of L-ornithine and L-aspartic acid, improves the feeling of fatigue in flushers, but not in non-flushers, when waking up the morning after drinking [13]. Although the study clearly showed the effectiveness of L-ornithine-L-aspartate, the outcome measures depended on subjective evaluations alone. Furthermore, it remained unclear whether the observed effects were predominantly caused by ornithine or by aspartic acid, because L-ornithine-L-aspartate contains equivalent amounts of ornithine and aspartic acid. In the present study we performed two randomized, placebo-controlled, double-masked cross over experiments using flushers as subjects. In experiment 1, to objectively examine the effects of 400 mg ornithine on the negative effects caused by alcohol consumption, we measured the typical salivary stress markers cortisol, secretory immunoglobulin A (sIgA), and α-amylase in addition to self-reported subjective feelings the morning after drinking. Furthermore, we investigated both the subjective and physiological effects of ornithine on acute ethanol metabolism in experiment 2.

## Methods

### Subjects

Subjects were recruited voluntarily from employees of Kirin Holdings to participate in the study. They were healthy Japanese adults with ‘flusher’ type alcohol tolerability. The study population was composed of 10 males and 1 female in experiment 1 (age, 35.5 ± 2.4 years; body weight, 65.4 ± 3.6 kg), and 11 males and 5 females in experiment 2 (age, 33.7 ± 1.9 years; body weight, 60.4 ± 2.7 kg). Subjects were free of medication and had no medical history of serious illness, alcohol abuse, or insomnia. All subjects signed an informed consent form after sufficient explanation of the purpose and design of this study. This study was approved by the Kirin Holdings Ethics Committee and carried out in accordance with the ethical principles of the Declaration of Helsinki.

### Alcohol patch test

Alcohol sensitivity was assessed using an alcohol patch test kit (Life Care Giken, Toyama, Japan). The patch was attached to the skin of the inner upper arm for 20 min and then removed. Subjects whose skin showed erythema in the patched area were judged to be flushers.

### Questionnaires

A visual analog scale (VAS), the Oguri-Shirakawa-Azumi sleep inventory MA version (OSA-MA), a profile of mood states (POMS), and a drunkenness feeling test were used in this study. The VAS used in this study was composed of 7 questions regarding sleepiness, awareness, fatigue, lassitude, sleeping contentment, deep sleep, and falling asleep. In each of the questions subjects were instructed to mark a spot on a 100 mm straight line according to their feelings upon awakening (left end 0 = best, right end 100 = worst). The distance of the mark from the left end was measured and used as the VAS score. A low VAS score indicates a good mood.

The OSA-MA is a self-report questionnaire composed of 16 items with a 4-point scale, which are consolidated into “sleepiness on rising”, “initiation and maintenance of sleep”, “frequent dreaming”, “refreshing”, and “sleep length” subscales [14]. A high OSA-MA subscale score indicates better sleep state.

The POMS test (brief version) is composed of 30 questions with a 5-point scale about the current mood state, which are classified into “tension-anxiety”, “depression-dejection”, “anger-hostility”, “vigor”, “fatigue”, and “confusion” subscales [15]. A low POMS score indicates better mood state except for the “vigor” factor.

The drunkenness feeling test was designed to measure subjective feelings of typical symptoms after alcohol drinking. It was composed of 5 questions. With a 9-point scale, regarding drunkenness, elation, swaying, weakness, and discomfort.

### Saliva collection and measures

Saliva samples were collected by using cotton swabs in Salivette devices (Salimetrics, State College, PA). Subjects were instructed to refrigerate sample tubes after collection and submit them to the laboratory on the morning of the study day. Sample tubes were immediately centrifuged at 1,500 × g for 10 min to collect the saliva from the swab. Saliva samples were stored at −80°C until analysis. Cortisol, sIgA and α-amylase concentrations were determined by assay kits according to the manufacturer’s instructions (Salimetrics).

### Breath collection and measures

Subjects exhaled completely into Tedlar® gas sampling bags (As One, Tokyo, Japan). Ethanol concentrations of the breath samples were immediately assessed by ethanol detector tubes (Gastec, Kanagawa, Japan).

### One-leg standing time

Subjects were instructed to stand on one leg with their eyes closed, with the contralateral knee flexed slightly and the hands placed at the waist. Time before falling over was measured and the average time of 3 repetitions was determined.

### Experimental design

The study consisted of two randomized, placebo-controlled, double-masked crossover experiments. Subjects were asked to take capsules containing ornithine or placebo during each experiment. The order of supplements was randomly determined for each subject and kept secret by the independent party until the data validation was completed, so that both subjects and investigators involved in the experiment were unaware of each subject’s assignment. The placebo capsules contained microcrystalline cellulose (Asahi Kasei Chemicals, Tokyo, Japan), and the ornithine capsules contained 500 mg L-ornithine monohydrochloride (Kyowa Wellness, Tokyo, Japan), which contains 400 mg ornithine. The two types of capsule were identical in appearance. Subjects were allowed free access to water, except during a water fasting period, but were instructed to take similar amounts of water between the trials. To eliminate the effects of the test supplements and alcohol consumed in the first trial, the second trial was performed no less than 2 days later.

Experiment 1 was done in subjects’ homes. Subjects all ate the same dinner 3.5 h before their usual bed time and then rested for 2 h with no food and drink. Subjects took a saliva sample and then drank 0.4 g/kg body weight of alcohol served as beer (5% alcohol) over 0.5 h. Subjects took the capsules containing placebo or ornithine 0.5 h after drinking and went to bed after another 0.5 h. The next morning, saliva collection and questionnaires (VAS, OSA-MA and POMS) were completed just after awakening.

Experiment 2 was done in the laboratory for a maximum of 5 subjects in one day. Subjects fasted for 2 h from 3 pm, and then took the capsules. After taking the capsules, the subjects drank alcohol as in experiment 1. Subjects took the same capsules again 0.5 h after drinking and then rested for 2.5 h. They repeated the drunkenness feeling test, breath collection and one foot standing test before drinking and 0, 30, 60, 120 and 180 min after drinking.

### Statistical analysis

Data are expressed as mean ± SEM. The questionnaire scores of VAS, OSA-MA, and POMS and salivary stress markers were compared using two tailed parametric paired *t*-tests. Other parameters were compared using two-way analysis of variance for repeated measures. A *p*-value of < 0.05 was considered statistically significant.

## Results

In experiment 1, sleep duration did not significantly differ between placebo and ornithine study days (6.2 ± 0.3 and 6.1 ± 0.3 h, respectively), as subjects were asked to keep their usual daily rhythm. Mean VAS and POMS scores (except for “vigor”) were lower and OSA-MA and “vigor” POMS scores were higher after ornithine supplementation than after placebo. These changes indicate that ornithine supplementation resulted in a better subjective mood, with significant improvements in VAS scores for “awareness”, “feeling of fatigue”, and “lassitude”, the OSA-MA scores for “sleep length”, and the POMS scores for “anger-hostility” and “confusion” (Table 1).

**Table 1:** The effects of ornithine or placebo on VAS, OSA-MA, and POMS scores the morning after alcohol consumption (experiment 1)

		**Placebo**	**Ornithine**
VAS	Sleepiness	52.1 ± 5.1	38.6 ± 5.6
	Awareness	46.5 ± 5.7	32.6 ± 3.5*
	Feeling of fatigue	44.2 ± 4.8	30.1 ± 4.3*
	Lassitude	48.2 ± 5.4	35.7 ± 5.1*
	Sleeping contentment	40.8 ± 6.1	35.0 ± 5.6
	Deep sleep	40.0 ± 5.3	32.0 ± 5.3
	Falling asleep	34.3 ± 6.5	31.4 ± 6.0
OSA-MA	Sleepiness on rising	41.3 ± 2.0	44.0 ± 1.6
	Initiation and maintenance of sleep	45.4 ± 3.2	48.8 ± 1.9
	Frequent dreaming	48.1 ± 2.5	52.9 ± 2.1
	Refreshing	47.0 ± 2.1	49.7 ± 1.3
	Sleep length	38.0 ± 2.2	44.0 ± 2.3*
POMS	Tension-Anxiety	41.2 ± 2.6	38.4 ± 2.2
	Depression-Dejection	44.4 ± 2.4	43.5 ± 2.1
	Anger-Hostility	41.6 ± 1.0	39.5 ± 1.5*
	Vigor	39.3 ± 2.4	44.9 ± 2.2
	Fatigue	46.3 ± 2.9	41.2 ± 1.3
	Confusion	55.0 ± 2.9	47.8 ± 1.5*

*p < 0.05 compared with placebo.

**Figure 1:**
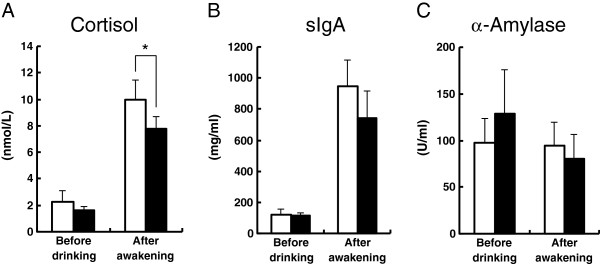
**Salivary concentrations of (A) cortisol, (B) sIgA, and (C) α-amylase before drinking and after awakening.** Open bars indicate control and closed indicate ornithine. Values are mean ± SEM, *p < 0.05.

In addition to the subjective feelings, salivary cortisol at the time of awakening was significantly decreased by ornithine administration (Figure 1). There were no significant changes in salivary sIgA or amylase concentrations.

To evaluate the effects of ornithine on acute ethanol metabolism, we investigated the effects of ethanol intake on the subjective feeling of drunkenness, on breath ethanol concentrations and on one-leg standing time in experiment 2. Intake of ethanol clearly affected both subjective and physiological states immediately after drinking. There were no significant differences between placebo and ornithine in any of the parameters (Table 2).

**Table 2:** Breath alcohol content, one-leg standing time, and drunkenness feeling test scores before and after drinking alcohol (experiment 2)

		**Before**	**Time after drinking (min)**				
	**Condition**	**drinking**	**0**	**30**	**60**	**120**	**180**
Breath alcohol content	Placebo	0.0 ± 0.0	458.3 ± 60.9	320.9 ± 36.2	217.2 ± 18.0	106.3 ± 11.0	48.8 ± 6.0
(ppm)	Ornithine	0.0 ± 0.0	437.7 ± 53.8	290.9 ± 25.0	216.0 ± 14.9	103.8 ± 9.5	45.3 ± 5.1
One-leg standing time	Placebo	40.6 ± 4.3	25.3 ± 4.7	25.0 ± 3.8	29.8 ± 4.6	34.5 ± 4.7	40.4 ± 5.0
(s)	Ornithine	41.5 ± 5.2	27.3 ± 3.9	23.2 ± 4.4	24.1 ± 3.9	32.3 ± 4.4	37.4 ± 4.6
Drunkenness feeling test							
Drunkenness	Placebo	1.0 ± 0.0	7.8 ± 0.3	5.8 ± 0.6	5.2 ± 0.5	4.1 ± 0.5	2.8 ± 0.3
	Ornithine	1.0 ± 0.0	7.4 ± 0.3	6.3 ± 0.5	4.9 ± 0.5	4.4 ± 0.5	3.3 ± 0.5
Elation	Placebo	1.8 ± 0.4	7.1 ± 0.4	5.5 ± 0.5	4.8 ± 0.5	3.9 ± 0.5	2.8 ± 0.5
	Ornithine	1.9 ± 0.3	6.8 ± 0.4	5.6 ± 0.4	5.1 ± 0.5	4.3 ± 0.5	3.1 ± 0.5
Swaying	Placebo	1.1 ± 0.1	5.3 ± 0.6	3.8 ± 0.7	3.0 ± 0.6	2.4 ± 0.4	2.0 ± 0.4
	Ornithine	1.0 ± 0.0	5.1 ± 0.6	3.4 ± 0.6	3.2 ± 0.6	2.5 ± 0.5	2.5 ± 0.5
Weakness	Placebo	1.8 ± 0.4	3.3 ± 0.7	3.6 ± 0.6	3.4 ± 0.6	2.8 ± 0.5	2.1 ± 0.4
	Ornithine	1.7 ± 0.3	3.5 ± 0.6	3.4 ± 0.8	3.3 ± 0.6	2.8 ± 0.5	2.2 ± 0.5
Discomfort	Placebo	1.4 ± 0.3	1.5 ± 0.3	1.5 ± 0.3	1.6 ± 0.2	2.1 ± 0.4	1.7 ± 0.3
	Ornithine	1.1 ± 0.1	1.8 ± 0.4	1.4 ± 0.3	1.4 ± 0.3	1.8 ± 0.4	1.4 ± 0.1

## Discussion

We evaluated the effects of ornithine on the alcohol-induced deterioration of mood states and on salivary stress markers the morning after alcohol intake in experiment 1 and on acute ethanol metabolism in experiment 2. Although flushers only constitute approximately 35% of the Japanese population [16,17], we chose to only use flushers as our study subjects. In a previous study we showed that ornithine-aspartate supplementation ameliorated alcohol-derived feelings of fatigue in flushers but not in non-flushers using almost the same study design as in the present study [13]. To be able to observe the effects of ethanol to the same degree, we decided to only screen flushers in this study. Previous studies by other groups generally used larger amounts of alcohol (0.7 to 1.6 g/kg body weight) [18-20], but the amount of alcohol consumed by flushers in our previous study (0.4 g/kg body weight) was considered appropriate to evoke the negative effects of alcohol the next morning.

In experiment 1, the subjective VAS factors “awareness”, “feeling of fatigue”, and “lassitude”, the OSA-MA factor “sleep length”, and the POMS factors “anger-hostility” and “confusion” the morning after drinking were significantly improved by ornithine intake. As all subjective factors tended to be improved by ornithine intake; alcohol-derived negative feelings seemed to be improved. Considering the fact that the OSA-MA factor “sleep length” was improved but substantive sleep time was not, ornithine made the subjects feel subjectively that they slept longer, even though the actual duration was the same.

Furthermore, the salivary stress marker cortisol was significantly reduced by ornithine intake. These results suggest that taking 400 mg of ornithine after alcohol consumption improves the mood on awakening, which is confirmed by the decrease in an objective stress marker.

In experiment 2 we aimed to clarify the influence of ornithine on acute alcohol metabolism. The feeling of drunkenness and other subjective and physiological symptoms of alcohol consumption were not significantly altered by ornithine intake. Thus, ornithine has little influence on acute alcohol metabolism at this dosage and the effect of ornithine on the alcohol-derived mood state the next morning was not caused by increased alcohol metabolism. Ornithine must therefore affect mood and physical state the next morning through other mechanisms. One of the possibilities may be through ammonia metabolism. Ammonia metabolism is disturbed by alcohol consumption [10], but is stimulated by ornithine administration [5,6]. Furthermore, there is a correlation between decreased ammonia metabolism and fatigue [21,22]. Thus, we speculate that ornithine may improve subjective feelings like “feeling of fatigue” by correcting the imbalance in ammonia metabolism caused by alcohol consumption. Further extensive studies are needed to clarify these associations.

Another mechanism behind the effects of ornithine on mood states after alcohol consumption may be through the improvement in sleep quality. Low amounts of alcohol can aid falling asleep, whereas higher amounts of alcohol cause sleep disturbances including frequent awakening and impaired normal sleep patterns. Omori et al. reported that oral administration of ornithine increased the amount of non-rapid eye movement (NREM) sleep in mice without changing the amount of rapid eye movement (REM) sleep [23]. In addition, intracerebroventricular injection of ornithine has a sedative effect on neonatal chicks, and ornithine can serve as a precursor for glutamate and proline, which have also been reported to have sedative or hypnotic effects [24-26]. Thus, the effect of ornithine on sleep length may be partially mediated by improvements in the disturbed sleep patterns caused by alcohol consumption.

Along with the subjective parameters, ornithine also improved salivary concentrations of cortisol. Cortisol is used in many studies as an indicator of the stress response of the hypothalamic-pituitary-adrenal (HPA) axis [27-30], which is activated by exposure to an acute stressor. A previous report has shown that oral administration of ornithine in mice reduced the stress-induced corticosterone levels [31]. Thus, ornithine may improve stress on awakening through suppression of the HPA axis.

There were some limitations with respect to the analysis, which may affect the accuracy of the results. The sample size was not sufficient to lead to a firm conclusion (experiment 1; n = 11, experiment 2; n = 16), and the proportion of female subjects was notably low in experiment 1. More extensive studies are needed to confirm our findings.

We investigated the effects of ornithine on the alcohol-derived adverse effects on subjective feelings and salivary markers the morning after alcohol consumption and on acute alcohol metabolism. Ornithine improved various negative feelings and decreased the salivary stress marker cortisol, but did not affect acute alcohol metabolism. Our results show that intake of 400 mg ornithine after alcohol consumption improved feelings of stress and fatigue upon awakening the next morning. Ornithine may be a potential dietary supplement for attenuating the morning after adverse psychological and physiological effects of alcohol.

## Competing interests

In this study, we used L-ornithine monohydrochloride, a product of Kyowa Wellness Company, Limited. This company is an affiliate of Kirin Holdings Company, Limited, to which the authors belong. The authors declare that no other financial support or compensation has been received for the study.

## Authors’ contributions

TKo conceptualized and carried out the study, performed the statistical analysis, and drafted the manuscript. EI assisted with the study and performed the statistical analysis. TKi conceptualized and coordinated the study. YM, MH and AT conceptualized the study and drafted the manuscript. All authors read and approved the final manuscript.

## References

[B1] Uchisawa H Sato A Ichita J Matsue H Ono T Influence of low-temperature processing of the brackish-water bivalve, Corbicula japonica, on the ornithine content of its extract Biosci Biotechnol Biochem 2004 68 1228 1234 10.1271/bbb.68.1228 15215585

[B2] Antoine FR Wei CI Littell RC Quinn BP Hogle AD Marshall MR Free Amino Acids in Dark- and White-muscle Fish as Determined by O-phthaldialdehyde Precolumn Derivatization J Food Sci 2001 66 72 77 10.1111/j.1365-2621.2001.tb15584.x

[B3] Frau M Massanet J Rosselló C Simal S Cañellas J Evolution of free amino acid content during ripening of Mahon cheese Food Chem 1997 60 651 657 10.1016/S0308-8146(97)00051-4

[B4] Morris SM Jr Regulation of enzymes of the urea cycle and arginine metabolism Annu Rev Nutr 2002 22 87 105 10.1146/annurev.nutr.22.110801.140547 12055339

[B5] Krebs HA Hems R Lund P Accumulation of amino acids by the perfused rat liver in the presence of ethanol Biochem J 1973 134 697 705 4749270 10.1042/bj1340697 PMC1177866

[B6] Staedt U Leweling H Gladisch R Kortsik C Hagmuller E Holm E Effects of ornithine aspartate on plasma ammonia and plasma amino acids in patients with cirrhosis. A double-blind, randomized study using a four-fold crossover design J Hepatol 1993 19 424 430 10.1016/S0168-8278(05)80553-7 8151104

[B7] Sugino T Shirai T Kajimoto Y Kajimoto O L-ornithine supplementation attenuates physical fatigue in healthy volunteers by modulating lipid and amino acid metabolism Nutr Res 2008 28 738 743 10.1016/j.nutres.2008.08.008 19083482

[B8] Ong JP Oehler G Kruger-Jansen C Lambert-Baumann J Younossi ZM Oral L-ornithine-L-aspartate improves health-related quality of life in cirrhotic patients with hepatic encephalopathy: an open-label, prospective, multicentre observational study Clin Drug Investig 2011 31 213 220 10.2165/11586700-000000000-00000 21208014

[B9] Cascales C Cascales M Santos-Ruiz A Effect of chronic ethanol or acetaldehyde on hepatic alcohol and aldehyde dehydrogenases, aminotransferases and glutamate dehydrogenase Rev Esp Fisiol 1985 41 19 27 2860705

[B10] Rejniuk VL Schafer TV Ivnitsky JJ Ammonia potentiates the lethal effect of ethanol on rats Bull Exp Biol Med 2008 145 741 743 10.1007/s10517-008-0191-6 19110566

[B11] Maier KP Volk B Hoppe-Seyler G Gerok W Urea-cycle enzymes in normal liver and in patients with alcoholic hepatitis Eur J Clin Invest 1974 4 193 195 4365846 10.1111/j.1365-2362.1974.tb00391.x

[B12] Goedde HW Harada S Agarwal DP Racial differences in alcohol sensitivity: a new hypothesis Hum Genet 1979 51 331 334 511165 10.1007/BF00283404

[B13] Kokubo T Ikeshima E Komatsu M Kirisako T Miura Y Horiuchi M Tsuda A L-ornithine-L-aspartate improves alcohol-derived fatigue feeling in flushers -A questionnaire study-Jpn Pharmacol Ther 2012 40 205 212

[B14] Yamamoto YTH Takase M Yamazaki K Shirakawa S Azumi K Standardization of revised version of OSA sleep inventory for middle age and aged Brain Sci Mental Disorders 1999 10 401 409

[B15] McNair D Lorr M Dropleman L:Profile of Mood States 1981 San Diego: Educational and Industrial Testing Service

[B16] Suzuki K Matsushita S Ishii T Relationship between the flushing response and drinking behavior among Japanese high school students Alcohol Clin Exp Res 1997 21 1726 1729 10.1111/j.1530-0277.1997.tb04515.x 9438538

[B17] Tsutaya S Shoji M Saito Y Kitaya H Nakata S Takamatsu H Yasujima M Analysis of aldehyde dehydrogenase 2 gene polymorphism and ethanol patch test as a screening method for alcohol sensitivity Tohoku J Exp Med 1999 187 305 310 10.1620/tjem.187.305 10503602

[B18] Collins WE Schroeder DJ Gilson RD Guedry FE Jr Effects of alcohol ingestion on tracking performance during angular acceleration J Appl Psychol 1971 55 559 563 5116652 10.1037/h0031868

[B19] Finnigan F Hammersley R Cooper T An examination of next-day hangover effects after a 100 mg/100 ml dose of alcohol in heavy social drinkers Addiction 1998 93 1829 1838 10.1046/j.1360-0443.1998.931218298.x 9926571

[B20] Stephens R Ling J Heffernan TM Heather N Jones K A review of the literature on the cognitive effects of alcohol hangover Alcohol Alcohol 2008 43 163 170 10.1093/alcalc/agm160 18238851

[B21] Wilkinson DJ Smeeton NJ Watt PW Ammonia metabolism, the brain and fatigue; revisiting the link Prog Neurobiol 2010 91 200 219 10.1016/j.pneurobio.2010.01.012 20138956

[B22] Mutch BJ Banister EW Ammonia metabolism in exercise and fatigue: a review Med Sci Sports Exerc 1983 15 41 50 6341752

[B23] Omori K Kagami Y Yokoyama C Moriyama T Matsumoto N Masaki M Nakamura H Kamasaka H Shiraishi K Kometani T Promotion of non–rapid eye movement sleep in mice after oral administration of ornithine Sleep Biol Rhythms 2012 10 38 45 10.1111/j.1479-8425.2011.00515.x

[B24] Yamane H Tsuneyoshi Y Denbow DM Furuse M N-Methyl-D-aspartate and alpha-amino-3-hydroxy-5-methyl-4-isoxazolepropionate receptors involved in the induction of sedative effects under an acute stress in neonatal chicks Amino Acids 2009 37 733 739 10.1007/s00726-008-0203-x 19018608

[B25] Hamasu K Shigemi K Tsuneyoshi Y Yamane H Sato H Denbow DM Furuse M Intracerebroventricular injection of L-proline and D-proline induces sedative and hypnotic effects by different mechanisms under an acute stressful condition in chicks Amino Acids 2010 38 57 64 10.1007/s00726-008-0204-9 19023642

[B26] Suenaga R Yamane H Tomonaga S Asechi M Adachi N Tsuneyoshi Y Kurauchi I Sato H Denbow DM Furuse M Central L-arginine reduced stress responses are mediated by L-ornithine in neonatal chicks Amino Acids 2008 35 107 113 10.1007/s00726-007-0617-x 18219550

[B27] Stratakis CA Chrousos GP Neuroendocrinology and pathophysiology of the stress system Ann N Y Acad Sci 1995 771 1 18 10.1111/j.1749-6632.1995.tb44666.x 8597390

[B28] Hellhammer DH Wust S Kudielka BM Salivary cortisol as a biomarker in stress research Psychoneuroendocrinology 2009 34 163 171 10.1016/j.psyneuen.2008.10.026 19095358

[B29] Buchanan TW Bagley SL Stansfield RB Preston SD The empathic, physiological resonance of stress Soc Neurosci 2011 7 191 201 21777106 10.1080/17470919.2011.588723

[B30] McGraw LK Out D Hammermeister JJ Ohlson CJ Pickering MA Granger DA Nature, correlates, and consequences of stress-related biological reactivity and regulation in Army nurses during combat casualty simulation Psychoneuroendocrinology 2012 38 135 144 22710003 10.1016/j.psyneuen.2012.05.009

[B31] Kurata K Nagasawa M Tomonaga S Aoki M Morishita K Denbow DM Furuse M Orally administered L-ornithine elevates brain L-ornithine levels and has an anxiolytic-like effect in mice Nutr Neurosci 2012 14 243 248 22053755 10.1179/1476830511Y.0000000018

